# A Two-Step Phenotypic Parameter Measurement Strategy for Overlapped Grapes under Different Light Conditions

**DOI:** 10.3390/s21134532

**Published:** 2021-07-01

**Authors:** Yubin Miao, Leilei Huang, Shu Zhang

**Affiliations:** School of Mechanical Engineering, Shanghai Jiao Tong University, Shanghai 200240, China; 369453619@sjtu.edu.cn (L.H.); ivorytower.zs@sjtu.edu.cn (S.Z.)

**Keywords:** contour fitting, edge segmentation, grape feature extraction, deep learning

## Abstract

Phenotypic characteristics of fruit particles, such as projection area, can reflect the growth status and physiological changes of grapes. However, complex backgrounds and overlaps always constrain accurate grape border recognition and detection of fruit particles. Therefore, this paper proposes a two-step phenotypic parameter measurement to calculate areas of overlapped grape particles. These two steps contain particle edge detection and contour fitting. For particle edge detection, an improved HED network is introduced. It makes full use of outputs of each convolutional layer, introduces Dice coefficients to original weighted cross-entropy loss function, and applies image pyramids to achieve multi-scale image edge detection. For contour fitting, an iterative least squares ellipse fitting and region growth algorithm is proposed to calculate the area of grapes. Experiments showed that in the edge detection step, compared with current prevalent methods including Canny, HED, and DeepEdge, the improved HED was able to extract the edges of detected fruit particles more clearly, accurately, and efficiently. It could also detect overlapping grape contours more completely. In the shape-fitting step, our method achieved an average error of 1.5% in grape area estimation. Therefore, this study provides convenient means and measures for extraction of grape phenotype characteristics and the grape growth law.

## 1. Introduction

Phenotypic characteristics of grape particles are important indicators in grape water diagnosis, berry growth monitoring, and grape growth modeling. They are also an important basis for detecting the healthy growth of grapes and ensuring high-quality size control during their growing process [1,2,3]. Machine vision and its related advanced technology have great application prospects in modern fruit planting and phenotype detection [4]; for example, the machine vision method is applied to the segmentation of grape clusters in vineyards [5]. However, accurate detection of edge contours has become an urgent unsolved problem in the field of grape phenotypic feature detection due to the common overlaps of grapes in natural environment.

Many researchers have proposed various methods for contour detection and segmentation using traditional machine vision technology. Jin Yan et al. [6] used an improved Hough transform to segment the grapes that had low detection accuracy and could easily miss when grapes overlapped. Arman Arefi et al. [7] used a watershed algorithm to segment overlapping tomatoes; the recognition accuracy reached 96.36%, but more easily resulted in over-segmentation. Xiang Rong et al. [8] proposed an overlapping tomato-recognition method based on edge curvature analysis. The accuracy of slightly occluded overlapping tomatoes was 90.9%, but when the fruit occlusion rate was high, the accuracy of recognition effect was reduced. Wang Qiaohua et al. [9] used the characteristics of curvature angle and mutation point to extract sizes of grape particles. Lei Yan et al. [10] proposed a particle-segmentation method based on contour and ellipse fitting. This method had high detection accuracy for elliptical fruit, but when the contour of the fruit was too short, it was easy to cause over-segmentation. Chenglin Wang et al. [11] used wavelet transform and a Retinex-based image-enhancement algorithm to segment oranges with an accuracy of 97.2%, but it was difficult to segment overlapping fruits. The gPb-Owt-Ucm image segmentation method proposed by Arbeláez Pablo et al. [12] achieved a score of 74% on the Berkeley segmentation data set, but the algorithm ran slowly. Dollár Piotr et al. [13] proposed an edge-detection algorithm based on structured forest that worked well, but the detection contour was thick. Although these methods achieved good results on grape segmentation to some extent, there still exists some shortcomings and limitations: (1) The traditional segmentation process is always multi-stage, and the complicated pre-processing and post-processing steps are relatively inefficient. In addition, since most pre-processing and post-processing steps are not universal, when the environmental parameters and grape colors change, the parameters in the processing flow also need to be fine-tuned in order to achieve the best results. (2) General detection algorithms have weak resistance to false edges and noise in images under complex conditions, which affects the accurate detection of grapes. (3) The detected edges might be inconspicuous, discontinuous, or missing for overlapped grape particles.

With the rapid development of deep learning in recent years, neural networks make it possible to extract high-dimensional contour features of overlapped grapes under complex conditions. The current methods can be divided into two branches. The first branch detects contours based on local areas, such as N4-field [14], DeepEdge [15], and DeepContour [16]. Their core pipeline is extracting the pixel block with the pixel as the center, extracting features using CNN, comparing the features with real contours, and predicting the pixel-level probability of contours. The above algorithm can better identify the contour of objects, but it also has the disadvantages of being time-consuming and memory-intensive in local area sampling. The other branch is end-to-end contour detection, represented by HED [17], RCF [18], and CEDN [19]. Its main idea is directly predicting the probability of pixel-level contours using CNN. Such an algorithm is simple and efficient, and the detection accuracy is high.

## 2. Methodology

To better extract features from overlapped grapes, this paper proposes a method with two steps: initial contour detection using a CNN-based network, and contour refinement using prior boundary shape knowledge. These will be further introduced in Section 2.1 and Section 2.2.

### 2.1. Step One: Contour Detection

An HED network is proposed to solve the ambiguity problem of edge detection in automatic rich-image hierarchy learning [20]. It performs an end-to-end pixel-level prediction using a backbone of VGG16 that contains 13 convolutional layers. These layers are divided into five stages, with an individual pooling layer at the end. The last convolutional layer of each stage is connected to the side output layer that produces an expected edge-prediction map, and the final edge prediction map is the fusion of all stages.

**Figure 1:**
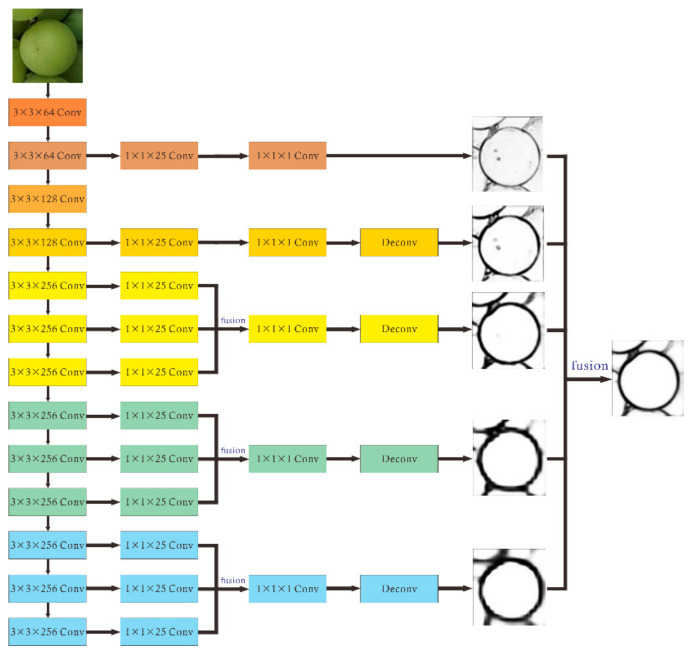
Structure of Improved HED network.

The above-mentioned original HED network can detect most edges in the image, but sometimes these edges are thick and not obvious, and some edges with low contrast might be missed, which might affect the accuracy of subsequent grape phenotypic parameter measurement. Therefore, this paper proposes three improvement measures to address these problems, as shown in Figure 1. First, more side output network structures are introduced in order to merge more edge-prediction maps at different levels and make it more conducive to extracting multi-scales features. Then, the Dice loss function [21] is added to the original weighted cross-entropy loss function. Finally, an image pyramid for multi-scale feature fusion is also introduced. Details of these adjustments are given below.

First, conventional HED only leads out the side output layer at the last convolutional layer of each stage, which does not make full use of the edge information contained in other convolutional layers. Inspired by RCF [18], we fused the resulting layers of VGG16 network in partial stages. Given the fact that our target scene was contour detection of grape particles, we paid more attention to high-dimensional feature maps to characterize the outline of the main part of the image, while low-dimensional feature maps are more suitable for characterizing image texture, noise, and other details. Therefore, compared to RCF, which accumulates the resulting layers in each stage using an eltwise layer to attain hybrid features, the improved HED network only connects all the convolutional layers in the third to fifth stages to the side output layers.

Taking the third stage as an example, each convolutional layer leads to a side output layer, the size of the convolution kernel is 1 × 1, and the channel depth is 25. The feature maps generated in each stage are superimposed and connected to a 1 × 1 − 1 convolutional layer. The edge-prediction image at this stage is obtained using upsampling that adopts a bilinear interpolation method to restore the predicted image to original size. The feature maps of all five stages are merged to predict the final edge map.

Second, most of the pixels in the image are non-edge pixels, and the distribution of edge/non-edge pixels is very unbalanced, so the general cross-entropy loss function used in classic two classification problems is difficult to adapt to such unbalance. To solve this problem, borrowed from [22], our loss function involves weighted cross-entropy loss:

(1) $$L(W,w)=-\beta \sum_{j\in Y_{+}}\log Pr(y_{j}=1|X;W,w)-(1-\beta )\sum_{j\in Y_{-}}\log Pr(y_{j}=0|X;W,w)$$

where $Y_{+}$ and $Y_{-}$ represent the collection of edge pixels and the collection of non-edge pixels in the picture, respectively. $\beta =\left|Y_{-}\right|/\left|Y\right|$, $1-\beta =\left|Y_{+}\right|/\left|Y\right|$, and $\left|Y\right|=\left|Y_{+}\right|+\left|Y_{-}\right|$ represent all pixels. *X* represents the input picture, and $Pr(y_{j}|X;W,w)$ is calculated by the pixel *j* through the Sigmoid function.

Weight coefficient β in Equation (1) is used to solve non-convergence in training caused by the unbalanced picture pixel distribution, but it might also cause unclear edges in prediction results. In order to solve the contradiction caused by the weight coefficient, the Dice coefficient is introduced as the loss function [22]:

(2) $$L(P,G)=Dist(P,G)=\frac{\sum_{i}^{N}p_{i}^{2}+\sum_{i}^{N}g_{i}^{2}}{2\sum_{i}^{N}p_{i}g_{i}}$$

where *G* is the actual edge map of the image, P is the predicted image, and $P_{i}$ and $g_{i}$ represent the pixel value of the *i*-th point in the predicted image and the actual edge image, respectively.

The Dice coefficient is used as a solution of unbalanced image pixel distribution because it defines a measure of pixel-level similarity between the edge-prediction image and ground truth. In this way, it supervises the model to converge to a high-quality outcome with thinner and more accurate boundaries. 

Considering the characteristics of the research object in this paper, a large number of single grape particles are used as data samples during the training process, and the effective edge pixels of the fruit occupy a small proportion of the whole picture. The problem of unbalanced image pixel distribution is more serious compared with other objects. Obviously, Dice loss is more suitable for our object. Therefore, the final loss of the model in this paper is the sum of weighted cross-entropy and the Dice loss:

(3) $$L(P,G)=\alpha L_{D}(P,G)+(1-\alpha )L_{w}(P,G)$$

where $L_{D}\left(P,G\right)$ represents the Dice loss function, and $L_{w}\left(P,G\right)$ represents the weighted cross-entropy loss function. 

**Figure 2:**
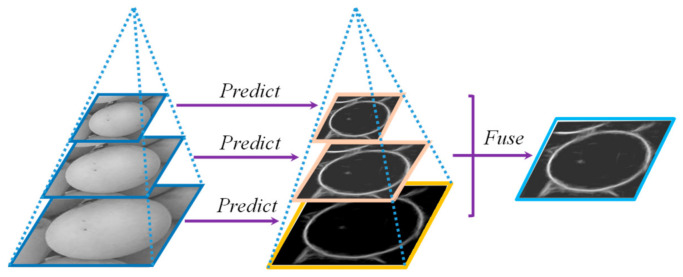
Schematic diagram of multi-scale contour prediction.

Third, grape particles usually exist in small-scale form in monitoring images, so image pyramids are used to enhance the model’s ability of representing grape contours at different scales. Specifically, the resolution of input image is adjusted to various levels using bilinear interpolation to form an image pyramid as network input. Then, different-size edge-probability maps are predicted by the network and resized to the same size as the input using bilinear interpolation upsampling. Finally, these images are fused to obtain the final prediction image. The whole process is illustrated in Figure 2.

### 2.2. Step Two: Contour Fitting

**Figure 3:**
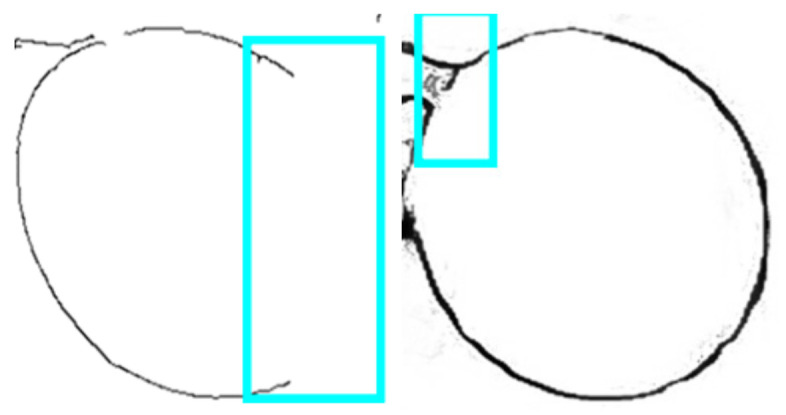
Grapes with missing contours.

**Figure 4:**
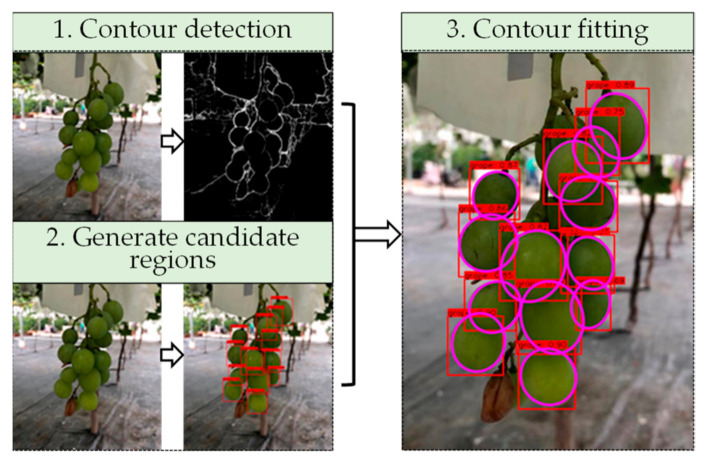
Grape contour fitting flowchart.

Under some poor environmental conditions, such as large overlapped areas or low light intensity, there might be quite large notches of contours, as shown in Figure 3. This would bring difficulties to further feature-extraction processes. Therefore, this paper proposes a new method based on grape contour fitting in candidate regions. As shown in Figure 4, based on the original image of the grape cluster, the contour extraction is performed, and target detection of fruit is performed to obtain candidate areas. After combining the contour map with the candidate area, each rectangular candidate area contains the contour of a grape. The grape contour fitting is performed on each candidate area in turn, so as to obtain the contour results of all grape particles. This method, combined with the improved HED network, forms our proposed two-step measuring strategy, which can detect and fit as many grapes as possible, and ensure a high-precision prediction of the long axis and the projected area of the fruit. 

#### 2.2.1. Candidate Region Generation

Learning-based object-detection methods can be divided into two-step or single-step methods. The representative method of two-step detection is Faster R-CNN, proposed by Ren et al. [23], while the representative method of single-step detection is YOLOv3 [24], which directly predicts targets from inputs and performs at a higher speed. Therefore, in this paper, YOLOv3 was used for candidate region generation.

#### 2.2.2. Iterative Least Squares Ellipse Fitting

There sometimes exist some noises, non-grape contours, or other grape contours in the new candidate area, and they might lead to biased outputs if all the sample data points are directly submitted into the calculation. In order to reduce the influence of error pixels on ellipse fitting, this paper proposes an iterative least squares ellipse fitting method based on the RANSAC algorithm. For a set of raw data points, they can be divided into two categories: inner points and outer points. Inner points are target data points, and their distribution can be described by a certain mathematical model. In this paper, inner points refer to outline pixels of the target grape. On the contrary, outer points such as noise, non-grape contour pixels, and other grape contour pixels are not applicable to the above mathematical models. The RANSAC algorithm was introduced to search for the best fit model that correctly clustered inner and outer points from iterative mathematical criterions. In this way, the proposed iterative least squares ellipse fitting can be summarized as follows:

(1) For the input edge contour map, record the edge contour pixels as *E*;
(2) Determine the number of iterations *K* as:
  (4) $$K=\frac{\log(1-p)}{\log(1-{\left(1-err\right)}^{s})}$$
  where p is the probability of a situation in which all sampling points are inner points in at least one of K iterations, err is the proportion of outer points among all data points, and s is the number of sample points selected for each sampling.
(3) Determine the evaluation criteria of model fitness:
  (5) $$fitness=\frac{N_{Dis\leq d}}{C}$$
  where C is the perimeter of ellipse obtained by fitting, and $N_{Dis\leq d}$ is the number of pixels in the image whose closest orthogonal distance to the ellipse is less than d.
(4) Randomly select 5 pixels to perform least squares ellipse fitting, and calculate the fit degree of the obtained ellipse fitting result, denoted as f. If the fit degree of the current model is higher than that of other models in the historical iteration process, record f as $f_{max}$.
(5) Repeat step 4, and loop *K* times to get the final ellipse equation.

**Figure 5:**
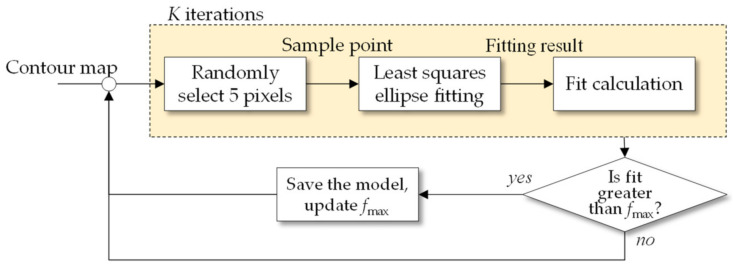
Flow chart of the iterative least squares ellipse fitting algorithm.

Figure 5 illustrates such calculation steps using a flow chart.

## 3. Experiments

The experiments were carried out in the greenhouse (31°11′ N, 121°29′ E) of the Modern Agricultural Engineering Training Center of Shanghai Jiao Tong University. In order to take pictures regularly, we developed an automatic image-acquisition system that included a digital camera with a maximum resolution of 3072 × 2304, a light source, and a timing controller.

**Figure 6:**
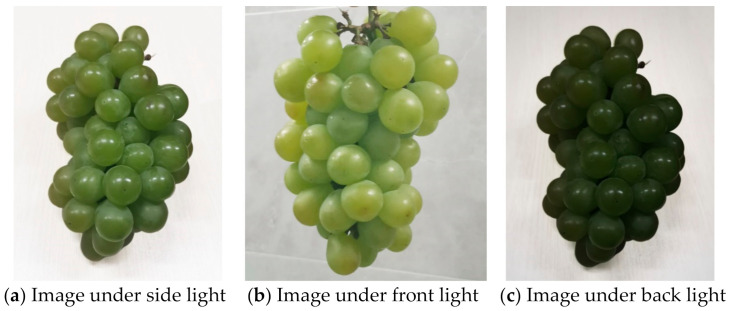
Images of Sunshine Muscat grapes under different lighting conditions.

Figure 6 shows images of Sunshine Muscat grapes under different lighting conditions: side light, front light, and back light. Among them, the pictures were continuously collected every hour during the expanding period, and at the color-changed period of the grapes in 10 days.

### 3.1. Grape Collection and Labeling

**Figure 7:**
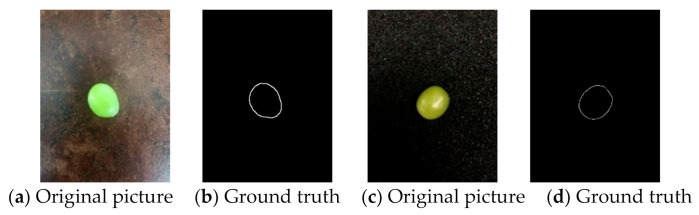
Original images and artificially labeled contour maps as ground truth.

A total number of 200 4032 × 3024-pixel photos of single Sunshine Muscat grapes under different backgrounds and environments were captured and uniformly reduced to 384 × 544 pixels, and the contours of the grapes were manually marked as ground truths, as shown in Figure 7. They were then augmented to 19,200 pictures after being sequentially flipped, rotated every 22.5°, expanded to 1.5 times, and educed to 0.5 times. 

### 3.2. Data Preparation for Object Detection

**Figure 8:**
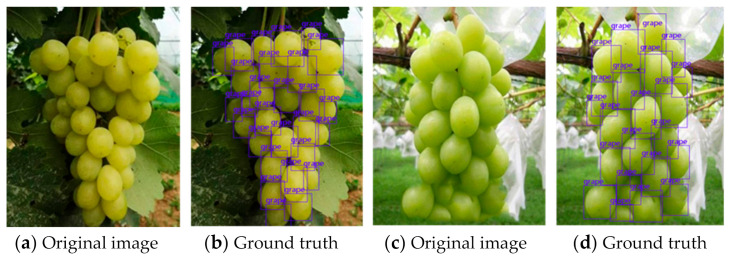
Grape clusters and their annotation maps.

The data set used in for object detection contained a total number of 300 pictures of grape clusters. In order to facilitate training, the sizes of the pictures were uniformly converted to 500 × 375 or 375 × 500, depending on their length and width. LabelMe [25] was used for annotation. The grapes that were not covered or the covered area that did not exceed about 80% were labeled, as shown in Figure 8.

## 4. Results and Discussion

### 4.1. Contour Detection Results

#### 4.1.1. Model Performance Comparison

Three criteria, optical dataset scale (ODS), optical image scale (OIS), and frame per second (FPS), were used as the model’s detection indicators. ODS refers to the detection score when all test set images used the same fixed threshold, and OIS refers to the detection score when the best threshold was used for each image in the test set. Before edge detection, it was necessary to perform non-maximum suppression (NMS) on the edge-prediction image, and then use Edge box [26,27,28] to perform testing.

**Figure 9:**
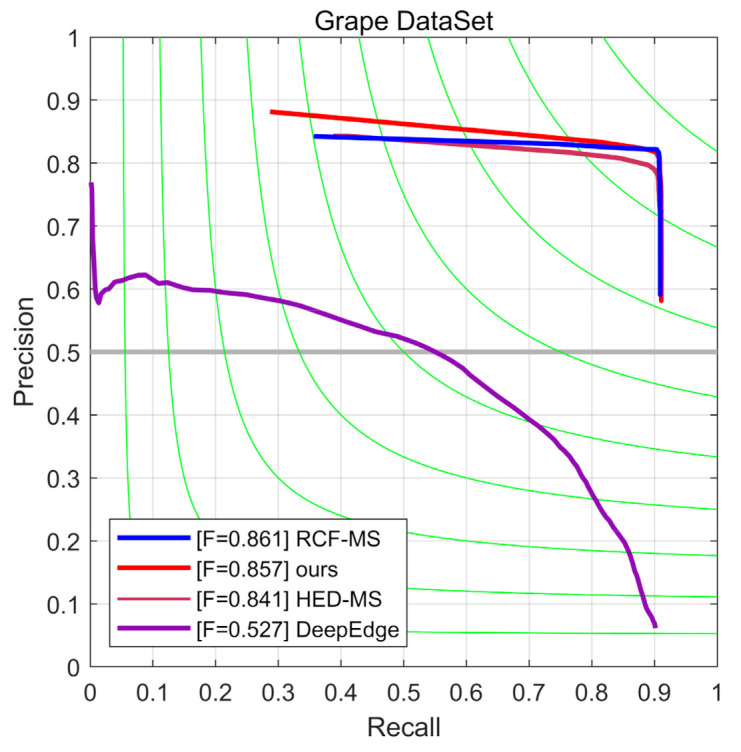
The evaluation results for the grape data set.

**Figure 10:**
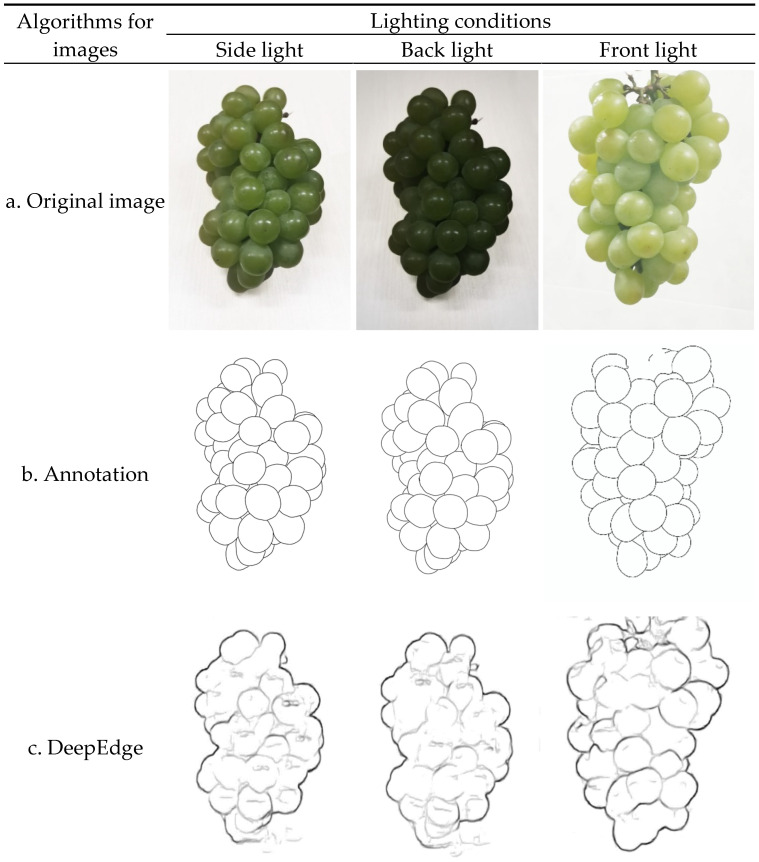

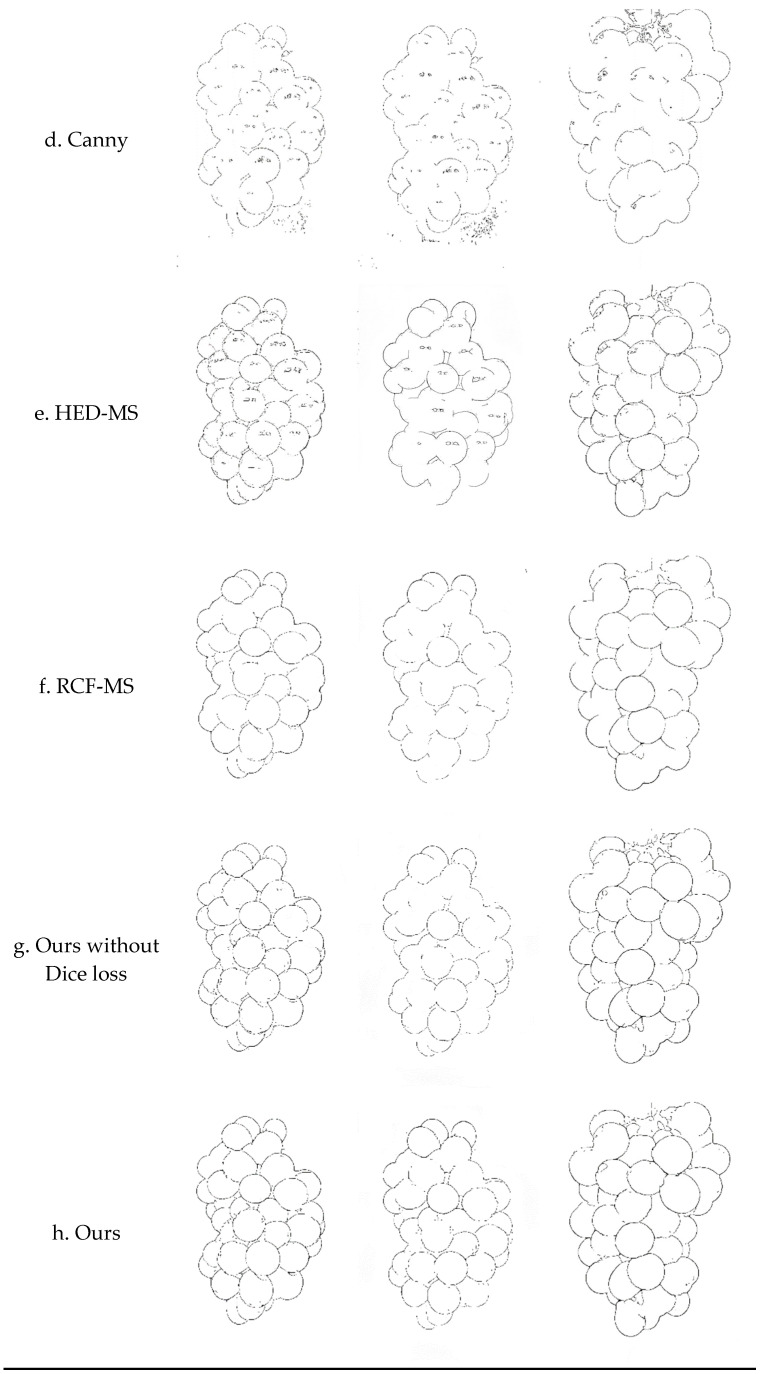
Experimental results of six algorithms for images with different lighting conditions.

In order to control the variables, the network adopted the optimized loss function, where α in Equation (3) is set to the optimal parameter value 0.6 determined by comparative experiments. Comparison results of our algorithm with DeepEdge, multi-scale version of HED, and multi-scale version of RCF are shown in Table 1 and Figure 9. We concluded that improved HED was higher than other models in terms of OIS and FPS. The reason why RCF outperformed our method in terms of ODS was because of the bias in the test data set, which consisted of much more individual grape particles than grape clusters. However, Figure 10 shows that improved HED could predict more complete contours of grape clusters.

#### 4.1.2. Comparison Test of Different Illumination Angles

In this section, the performances of our model are compared with Canny, HED-MS, DeepEdge, RCF-MS, and an ablated version without Dice loss. The following indicators were introduced to measure the effect of grape contour detection: Dice (*D*) coefficient, as the similarity between predicted contour map and ground truth; recall rate (*R*), as the ratio of the true examples in false-negative examples to the true examples in the predicted grape contour map; contour redundancy rate (*A*), as the ratio of the number of edge pixels in ground truth to that of all edge pixels in the predicted contour map, which reflects the model’s anti-interference ability against the contours or noise of objects other than grapes.

(6) $$D=\frac{2\left|X\cap Y\right|}{\left|X\right|+\left|Y\right|}$$

(7) $$R=\frac{TP}{TP+FN}$$

(8) $$A=1-\frac{TP}{Count(X)}\times 100\%$$

where X is the predicted map; Y is ground truth; *TP* is the number of edge pixels of the fruit that are correctly predicted; *FN* is the number of edge pixels of the fruit that are predicted as background pixels; and *Count*(*X*) is the total number of edge pixels in the output contour prediction image. In this section, a manual labeling method was used to evaluate the outline of grapes.

In natural environments, changes in lighting conditions will affect grape contour detection. In order to prove the adaptability of our method in various light conditions, we tested and compared the edge-detection abilities of different algorithms on grapes under front light, side light, and back light.

It can be seen in Figure 10 that the edges of the grapes are clear and easy to separate under the side light, and the surface of the grapes is uniformly illuminated, so the edge detection was easier. Under back-light conditions, the light intensity on surfaces of grapes was weak, and the contours were blurry. Under front-light conditions, some areas of grape surfaces formed bright white spots due to light reflection, and the contours of grapes were easy to confuse with the background. The contours of overlapped grapes detected by our improved HED network were more significant, the completeness of contours was higher under side-light conditions, and the unobstructed contours of grapes under back-light and front-light conditions were successfully recognized, although part of the contours of grapes blocked inside might have been missed.

Compared with HED, the contours of overlapped grapes detected by our algorithm were more complete, while HED had the error of recognizing reflections as contours. Furthermore, although RCF-MS might detect smoother contours of grapes, our method provided more complete contours for overlapped grapes under all three conditions, especially side light and back light. In addition, the integrity of contour detection was higher with the introduction of Dice loss, and the effect did not degrade much, even in the ablated version without Dice loss. In general, our algorithm was more comprehensive in contour detection of overlapped grapes.

It can be seen in Table 2 that the Dice coefficient of improved HED was more than 3% higher than other algorithms, and the recall rate was more than 2% higher than other algorithms. The redundancy rate of grape contours under different lighting conditions was only lower than RCF, and was higher than other algorithms.

### 4.2. Candidate Region Generation Results

**Figure 11:**
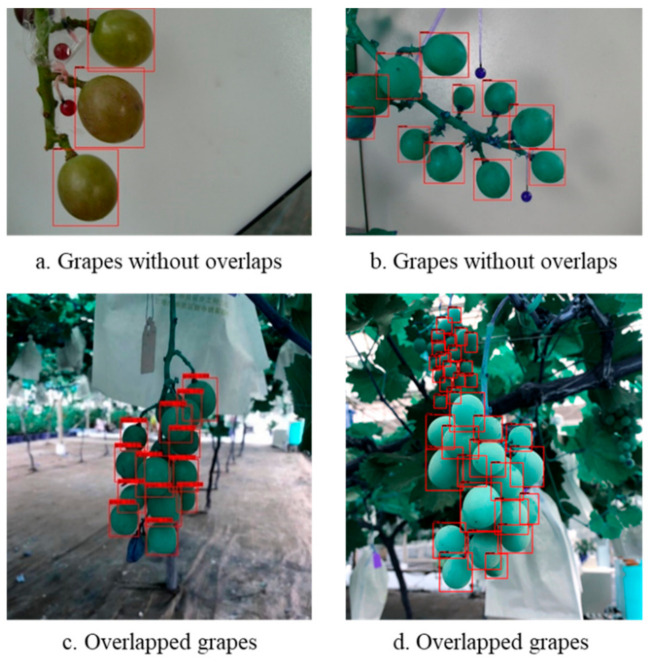
Detection of grape particles.

Figure 11a,b show the detection results of grape clusters without overlaps, while Figure 11c, show those with overlaps. It can be seen that most obscured and unobstructed grapes could be detected successfully. In addition, in Figure 11d, not only the grapes in the target area, but also some small-scale grapes in the background, were successfully detected. 

Tests were performed on a new set that contained 100 images of grape clusters. As shown in Table 3, YOLOv3 reached 95.44% in the F1 test for grapes, a 94.56% accuracy rate, and a 96.24% recall rate.

### 4.3. Contour-Fitting Results

#### 4.3.1. Recall Rate of Grape Contour Fitting

Recall rate defines the ratio of the number of successfully fitted grape contours to the actual number of grape contours, which is calculated as follows:

(9) $$R=\frac{N_{D}}{N_{A}}\times 100\%$$

where $N_{D}$ is the number of correct grape contours obtained by fitting, and $N_{A}$ is the actual number of grape contours in the image.

**Figure 12:**
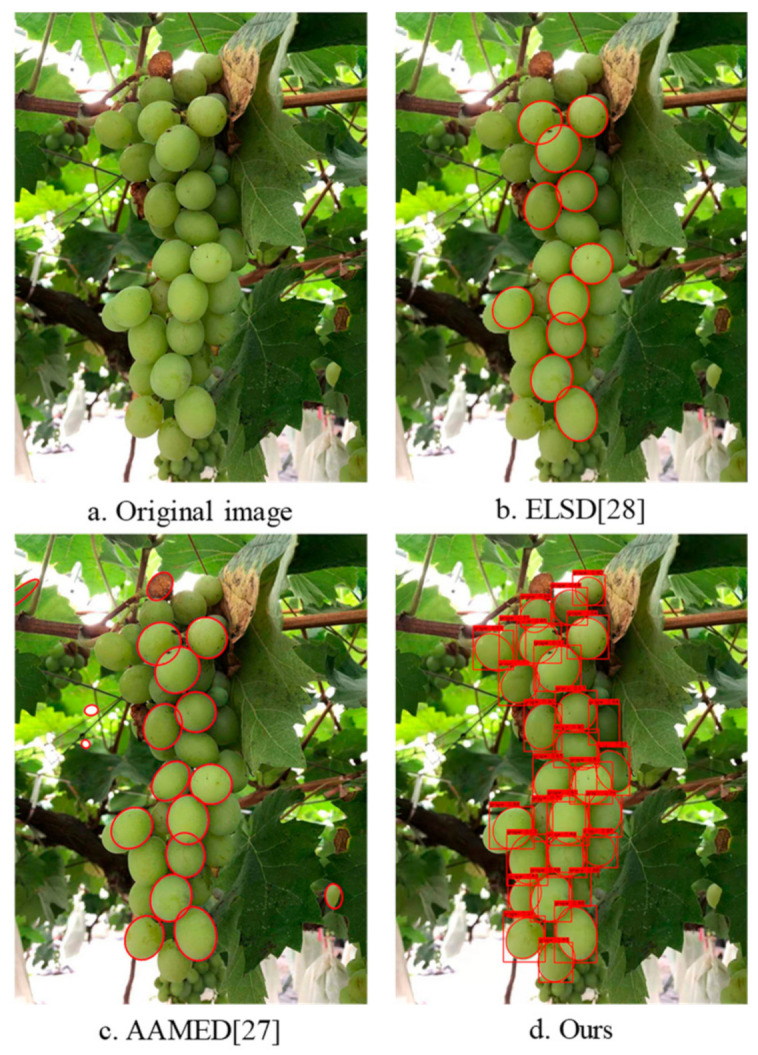
Test results of three fitting algorithms.

A total of 60 grapes with different clusters shapes and different environmental conditions were tested. Due to the growth characteristics of grapes, the fruit grains were mainly blocked by other adjacent fruit grains, and the cases of being blocked by branches and leaves rarely occurred. As shown in Figure 12, the heavily occluded grapes were ignored (most of the outlines of such grapes were not visible).

Three algorithms were used to detect 60 grape cluster images and calculate the average recall rate. As shown in Table 4, the recall rate of our method was higher than that of the other two algorithms.

#### 4.3.2. Contour-Fitting Accuracy Test

In this section, different grapes with manually made missing or noisy contours were used to verify the accuracy of our ellipse-fitting algorithm for grape particles.

**Figure 13:**
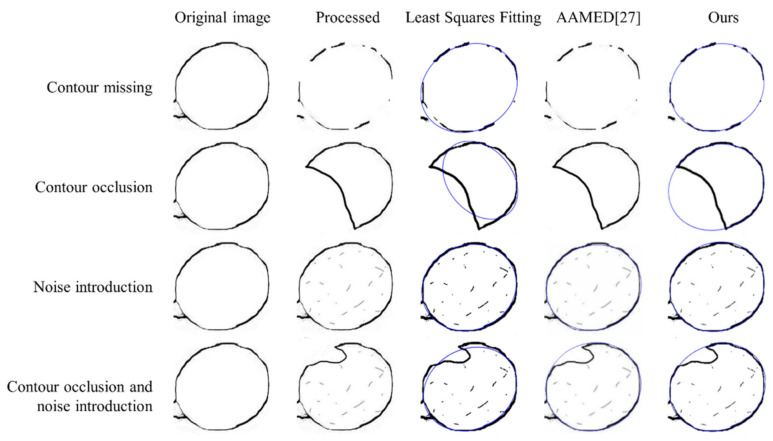
Grape contour-fitting results.

Results of fitting tests on 60 artificially processed grapes are shown in Figure 13; our method accurately simulated missing or noisy grape contours. The detection results showed that it had high accuracy for incomplete grape-grain contour fitting under a complex background, and a strong anti-interference ability of non-grape contour edges and noise. Table 5 shows that the error of long axis measurement of the artificially processed grape particle profile was about 1%. Table 6 shows that the error of projected area measurement of the artificially processed grape particle contour was within 1.1%, which met the actual application requirements. In these two tables, AARD means the average absolute relative deviation.

### 4.4. Continuous Monitoring of the Projected Area of Grapes

In this session, continuous monitoring of grapes cultivating was used as a practical verification of the effectiveness of our method, during which hourly and overall changes of projected areas of grapes were measured within six days during the expansion period. The instance-wise average was carried out over raw measurement results to reduce random errors.

**Figure 14:**
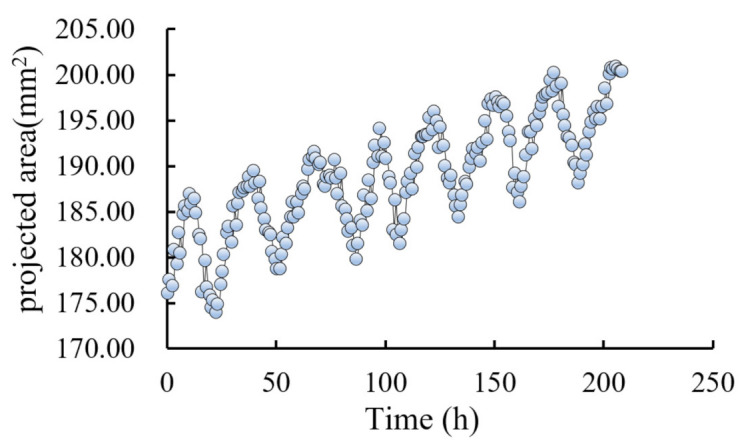
Changes of the projected area of the particles of Sunshine Muscat grapes over time.

**Figure 15:**
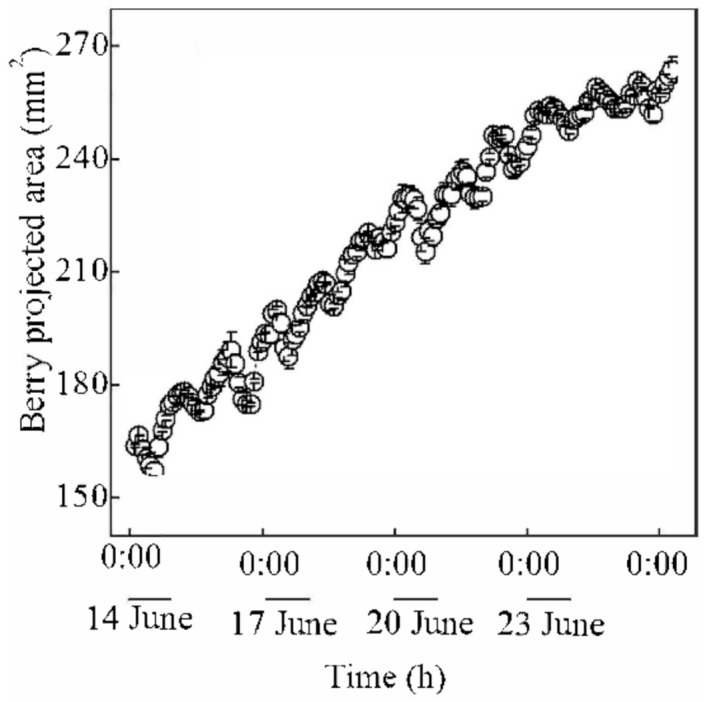
Theoretical curve of the change of the projection area of the berries of Sunshine Muscat grapes [29].

The measurement results are shown in Figure 14 and Figure 15. It can be seen that the projected area of grape fluctuated similar to a sinusoid curve every day, and the area showed an upward trend, which was consistent with the law that the projected area of grape fruit shrinks during the day and expands at night. It can be seen that the curve of projected area of fruit measured using our method was consistent with the theoretical law obtained in paper [29], which verified the effectiveness of our method. Furthermore, continuous monitoring of the projected area of grapes can be used for irrigation decision-making during grape fruit development [30].

## 5. Summary and Conclusions 

This paper proposed a two-step measurement strategy for contour analysis of overlapped grapes that consisted of edge detection and contour fitting. For particle contour detection, an improved HED network with weighted Dice loss was introduced. It made full use of internal features from each convolutional layer and applied image pyramids to achieve multi-scale edge detection. For contour fitting, a method that combined iterative least squares ellipse fitting and a region-growth algorithm was proposed to calculate the projected area of grapes. Experiments showed that in the edge-detection step, compared with current prevalent methods, our improved HED was able to detect and extract complete fruit particle edges more clearly, accurately, and efficiently. In the shape-fitting step, our method achieved an average error of 1.5% in grape area estimation. Therefore, we hope this study can provide convenient means and measures for grape phenotype characteristic extraction and the grape growth law.

## Figures and Tables

**Table 1:** Comparison of detection results of different network structures.

Network Structure	ODS	OIS	FPS
DeepEdge	0.527	0.571	8
HED-MS	0.841	0.853	32
RCF-MS	0.861	0.867	33
Ours	0.857	0.868	38

**Table 2:** Experimental results of five algorithms on grapes under different light conditions.

Light Conditions	Algorithms	Index		
		D	R	A
Side light	Ours	0.33	0.69	35.7%
	HED-MS	0.24	0.62	48.3%
	RCF	0.30	0.67	33.9%
	Canny	0.27	0.67	46.5%
	DeepEgde	0.20	0.42	41.6%
Back light	Ours	0.27	0.59	33.1%
	HED-MS	0.19	0.57	47.5%
	RCF-MS	0.23	0.54	31.5%
	Canny	0.21	0.49	43.2%
	DeepEgde	0.20	0.38	35.1%
Front light	Ours	0.31	0.63	32.3%
	HED-MS	0.22	0.59	43.3%
	RCF-MS	0.28	0.59	31.0%
	Canny	0.18	0.51	51.9%
	DeepEgde	0.24	0.47	34.2%

**Table 3:** Test results of grape fruit detection.

Index	F1/%	Precision/%	Recall/%
Score	95.44	94.65	96.24

**Table 4:** Recall test results.

Fitting Method	Recall/100%
Ours	96.34
AMMED	88.7
ELSD	81.5

**Table 5:** Measurement results of long axis of different grape particle contours by different algorithms.

Serial Number	The Least Square Method/Pixel	Aamed/Pixel	Elsd/Pixel	Ours/Pixel	Ground Truth/Pixel
1	530	537	560	542	545
2	545	540	540	548	550
3	589	595	595	599	603
4	600	587	590	591	593
5	570	570	571	576	577
6	564	561	561	563	569
7	553	573	569	575	573
8	575	587	584	578	581
9	573	579	570	573	573
10	555	562	560	570	569
AARD	1.62%	1.16%	1.21%	0.62%	

**Table 6:** Measurement results of the projected area of different grape particle contours by different algorithms.

Serial Number	The Least Square Method/Pixel	AAMED/Pixel	ELSD/Pixel	Ours/Pixel	Ground Truth/Pixel
1	304,285	315,765	305,894	315,951	318,959
2	342,628	347,598	342,146	347,561	345,793
3	274,105	289,075	274,589	289,014	288,606
4	220,553	216,800	201,454	216,859	219,015
5	238,936	242,457	245,896	241,367	242,047
6	199,784	204,784	203,654	205,657	203,955
7	55,334	55,697	54,356	53,738	54,728
8	39,128	42,475	40,289	39,418	40,242
9	54,456	53,457	53,006	53,982	53,287
10	57,055	57,689	56,126	56,529	56,224
AARD	2.21%	1.35%	2.12%	0.88%	

## Data Availability

Not applicable.
